# Feature Extraction Methods Proposed for Speech Recognition Are Effective on Road Condition Monitoring Using Smartphone Inertial Sensors

**DOI:** 10.3390/s19163481

**Published:** 2019-08-09

**Authors:** Frederico Soares Cabral, Hidekazu Fukai, Satoshi Tamura

**Affiliations:** 1 Faculty of Engineering, National University of East Timor, Hera, East Timor; 2 Faculty of Engineering, Gifu University, Gifu 501-1193, Japan

**Keywords:** road condition monitoring, paved and unpaved classification, smartphone inertial sensors, feature extraction, signal processing, deep neural network

## Abstract

The objective of our project is to develop an automatic survey system for road condition monitoring using smartphone devices. One of the main tasks of our project is the classification of paved and unpaved roads. Assuming recordings will be archived by using various types of vehicle suspension system and speeds in practice, hence, we use the multiple sensors found in smartphones and state-of-the-art machine learning techniques for signal processing. Despite usually not being paid much attention, the results of the classification are dependent on the feature extraction step. Therefore, we have to carefully choose not only the classification method but also the feature extraction method and their parameters. Simple statistics-based features are most commonly used to extract road surface information from acceleration data. In this study, we evaluated the mel-frequency cepstral coefficient (MFCC) and perceptual linear prediction coefficients (PLP) as a feature extraction step to improve the accuracy for paved and unpaved road classification. Although both MFCC and PLP have been developed in the human speech recognition field, we found that modified MFCC and PLP can be used to improve the commonly used statistical method.

## 1. Introduction

East Timor is a new country that gained independence in 2002. It is located in Southeast Asia, to the east of Indonesia and to the northwest of Australia. Being a developing country, the roads have become an important infrastructure for the country’s economic growth. East Timor has more than 6000 km of road networks including 1426 km of national roads, 869 km of district roads, 716 km of urban roads, and more than 3000 km of rural roads (unpaved) [1,2,3].

To provide a good quality of road, regular monitoring activities are a major challenge for transport infrastructure management. Generally, two types of approaches have been used to monitor road conditions: One is a traditional approach in which the road condition is inspected manually and the other approach uses some existing commercial products such as “Road Scanner” [4]. Being time-consuming and requiring human experience are disadvantages of the traditional approach; meanwhile, the high cost of commercial products is not practical for an underdeveloped country such as East Timor.

Recently, the use of multisensor technology in research activities has increased due to a decrease in sensor prices. One of the research areas that has increased rapidly is road condition monitoring using multisensor technology. A smartphone device is one of the solutions chosen by many researchers to monitor road conditions due to the number of smartphone users increasing every year around the world [5], including East Timor [6], and also such devices are equipped with various sensors, i.e., an accelerometer, gyroscope, compass, global positioning system (GPS), etc. An accelerometer and gyroscope sensor can be used to collect information for the road surface by fixing the smartphone on the dashboard of a moving vehicle, while a GPS sensor can be used to record the location. Various types of road conditions such as potholes, bumps, anomaly, paved, unpaved, and roughness estimation can be identified by analyzing acceleration and gyroscope signals.

The overall aim of our project is to build an integrated system that surveys road surface conditions using smartphone sensors [7]. However, due to the fact that almost 50% of the road networks in East Timor are unpaved rural roads, there is a strong requirement for the classification of paved and unpaved roads. In addition to the paved and unpaved road classification, road surface qualification by roughness estimation is essential to provide riding comfort and safety. Therefore, in this study, we focused on the classification of paved and unpaved roads, which becomes a preliminary task when conducting roughness estimation for both paved and unpaved roads.

For the problem of road condition classification, various methodologies have been proposed so far. Generally, the entire process of road condition classification is divided into several steps such as preprocessing, feature extraction and classification. After signal acquisition, rotation, denoising, smoothing, segmentation, and thresholding are the main operations in the preprocessing step. The next feature extraction step is a process that transforms a regular vector into a manageable vector that is suitable for use as an input for subsequent classification methods. In the last classification step, the road conditions are evaluated from their features by certain methods to predict the class of the given data points. Here, the feature extraction step is important for obtaining a better result at the classification step.

This paper studies the influence of feature extraction method to improve the performance of the classification of paved and unpaved road. The results show that the frequency-based feature extraction, i.e., MFCC and perceptual linear prediction (PLP) obtained better performance than statistics-based feature extraction.

The remaining part of the paper is structured as follows. Section 2 describes related works, while Section 3 describes the system architecture. In Section 4, the classification method, dataset, and performance evaluation are described. Section 5 presents the experimental results using training and validation dataset as well as testing dataset then followed by map visualization. Section 6 describes the discussion. Finally, we summarize our results in Section 7.

## 2. Related Works

### 2.1. Related Works on the Use of Inertial Sensors

Several studies have been proposed to use the accelerometer sensor for road condition monitoring. Eriksson et al. developed a system called “The Pothole Patrol” based on an accelerometer and GPS sensor to detect potholes and other road anomalies [8]. A system called “Nericell” was demonstrated by Mohan et al., which used several sensing components such as an accelerometer, a microphone, a GSM radio, and a GPS sensor to detect braking, stop-and-go traffic, and bumps [9]. An application of a machine learning technique, i.e., a support vector machine (SVM), was proposed to detect road anomalies using a smartphone device [10,11]. An accelerometer, a GPS, and a magnetometer in a smartphone device were used for breaking and bump detection using K-means clustering and a SVM technique [12]. Tomiyama et al. [13] investigated the use of a standalone accelerometer sensor for estimating road surface conditions based on the international roughness index (IRI) value. A collaborative system for monitoring road surface conditions was proposed by Alessandroni et al. [14]. In the study, an Android application called “SmartRoadSense” was built to compute the power of the prediction error $\left(P_{P_{E}}\right)$ value in real-time. The authors estimated the road roughness by taking the average of $\left(P_{P_{E}}\right)$ and visualized it with maps. Masino et al. proposed a real-time road damage detection system using an accelerometer sensor, an angular rate sensor and a GPS sensor [15]. A community sensor network for road roughness monitoring using a smartphone was proposed by Kumar et al. [16]. A fuzzy classifier has been used to classify roads into several categories, i.e., smooth, minor discomfort, moderate discomfort, major discomfort, and very rough [16]. The system involved a client application on a smartphone that collects acceleration data, processes the information and communicates to a central server followed by visualizing of the road conditions on Google Maps. Singh et al. proposed a system for detecting potholes and bumps was proposed by calculating the similarity of two signals using a dynamic time warping (DTW) technique [17].

Based on the above references, the majority of the studies have focused on the conditions of only a paved road, and, as far as we know, there is only one study that has discussed the classification of paved and unpaved roads using smartphone sensors [7]. However, in fact, the classification of paved and unpaved roads is still important in developing countries, and this is not an easy task because the vibration varies depending on the vehicle suspension type, vehicle speed, driving style, and so on. On the other hand, the vibration of a vehicle on rough paved roads is larger than that of smooth unpaved roads. Meanwhile, in the field of road condition monitoring, the feature extraction step has not been well discussed so far.

### 2.2. Related Work on Features Extraction from an Inertial Sensor

A feature is a distinctive or characteristic measurement, transform, or structural component extracted from a segment of a pattern, which can be used to minimize the loss of important information [18]. Feature extraction methods could be based on either calculating statistical characteristics or producing syntactic descriptions [18].

To deal with the classification/detection problem, many studies rely on feature extraction including the road condition monitoring field. As shown in Table 1, there are several relevant amounts of road condition monitoring works that apply feature extraction in different ways on different targets. For the features, most of these works are calculating statistical characteristics (mean, standard deviation, etc.) and common signal processing techniques (Fast Fourier-transform (FFT) and wavelets transform) to extract information from accelerometer signals.

In this study, we propose the use of advanced frequency-based features that are mostly used in speech recognition, i.e., mel-frequency cepstral coefficients (MFCC) and perceptual linear predictive (PLP) coefficient for feature extraction in a road condition monitoring task, especially for paved and unpaved road classification. In addition to the speech recognition field, both methods have been applied to other research areas including the HAR (human activity recognition) field [19,20,21].

In our previous work [7], we conducted paved and unpaved road classification based on statistics-based feature extraction, and we obtained more than 97% accuracy for the classification. However, when the dataset included new data that were recorded by a vehicle with soft suspension and at various speeds, we faced a decline in classification performance. Therefore, in this study, we tried to improve the classification results by focusing on the feature extraction steps.

## 3. System Architecture

**Figure 1:**
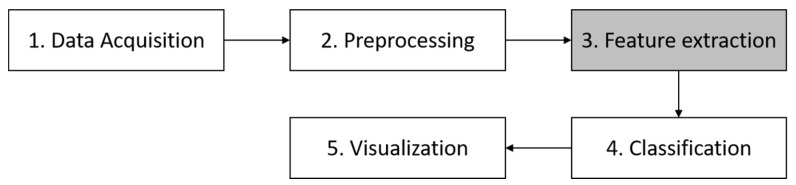
Overall system architecture for paved and unpaved road classification.

The overall system architecture for paved and unpaved road classification is shown in Figure 1. Basically, there are five steps in this architecture i.e., data acquisition, preprocessing, feature extraction, paved and unpaved road classification, and visualization. 

### 3.1. Data Acquisition

Road data were recorded using an Android application, which was developed in our laboratory. The application monitors 11 dimensions in total of the Android smartphone sensor data, i.e., three axis of the accelerometer and gyroscope, GPS, compass, and timestamp. The data were recorded at a sampling rate of 100 Hz for the accelerometer and gyroscope sensor, and at 1 Hz for the GPS sensor.

The data collection was conducted on the East Timor road network, including national roads, district roads, and rural roads. The road data were recorded on approximately 200 km of road and 32,264 data points were generated. The roads include various types, such as paved roads and unpaved roads, and features, such as potholes and bumps. The road data were collected using various types of smartphones and vehicles due to the assumption that the developed system will be used by local surveyors in practice. We used three different smartphone devices, i.e., Samsung Galaxy S8 (2), Samsung Galaxy A5 (1), and Asus ZenFone (1). To deal with the various types of vehicle suspension system, four types of vehicle were used, i.e., Toyota Land Cruiser Prado, Mitsubishi Pajero, Toyota Hi-Lux, and Daihatsu Xenia. We installed a data acquisition application, which we developed on each smartphone. The smartphone was fixed on the dashboard of each vehicle. For the verification purpose, we made recording with not only smartphones but also dash cam. The movies are used to check the actual road condition compared to the classification results using smartphone sensor data. In addition, we ordered the drivers to drive with the standard way of driving depends on the road condition.

### 3.2. Preprocessing

In practice, smartphones might be placed with various postures in the vehicle cabin. To overcome this situation, the Euler angle and a rotation matrix [17,22] are used to virtually reorient the angle of the smartphone. A third-order low-pass Butterworth filter [16] with a 5 Hz cut-off frequency was applied to remove undesired signals. The value of 5 Hz was chosen due to the minimum tolerance of the human body to vertical vibration being at 5 Hz according to the book [23].

### 3.3. Feature Extraction

This step is the main focus of this study. The objective is to find a suitable feature extraction method to improve accuracy for the classification of paved and unpaved roads. Usually, simple statistical features are used in a road surface monitoring system [7]. On the other hand, there are two well-known advanced feature extraction methods in speech signal processing, i.e., MFCC (mel-frequency cepstral coefficients) and PLP (perceptual linear predictive) coefficients. Each feature extraction method will be described in detail in Section 5.

### 3.4. Classification

In this step, the system classifies the road as paved or unpaved from the signal features by an appropriate classification method. The details are described in Section 4.

### 3.5. Visualization

**Figure 2:**
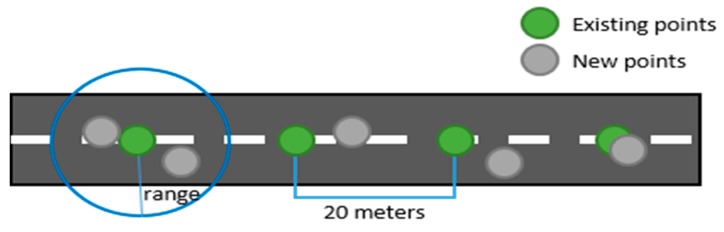
The map visualization process.

The classification results were visualized on a map provided by Google Maps. In the map, we used green points to indicate paved roads and red points to indicate unpaved roads. There are three steps in the visualization process (Figure 2). The first step is to make default points for the entire road at 20 m intervals. In the second step, the “snap to roads” technique of the Google Roads API is applied to match the GPS points to the center of the roads. The last step is aggregation, in which the closest distance between new points and existing points is calculated, and the existing points are updated by taking the median value in the range of 20 m. A MySQL database was used to store the data including the classification result, latitude, and longitude.

## 4. Classification, Dataset, and Evaluation

### 4.1. Support Vector Machine (SVM)

The SVM is a supervised learning method used for regression, classification, and outlier detection. Before the advent of deep learning techniques, SVM was among the most popular methods for machine learning classification and regression problems. Currently, SVM is still a powerful method, especially for road condition monitoring. In SVM, model selection and parameter search play a crucial role. It is known that the general performance of SVM depends on good settings of the kernel, regularization (often known as the *C* parameter) and gamma (γ). The kernel transforms data into another dimension that can be used to clearly define the margin between classes. The kernel can be linear, radial basis function, polynomial and sigmoid. The *C* parameter trades the misclassification of training examples for simplicity in the decision surface. The γ parameter defines how far the influence of a single training example reaches [24]. In addition, the SVM has another advantage, i.e., prediction less computationally expensive compared to other methods such as Deep Learning.

In this study, we applied the SVM model to conduct the classification task. As will be described in Section 4.2, there are two experiments. The first is feature extraction analysis and the second experiment involves a test dataset. Therefore, the parameter settings for SVM are shown in Table 2.

### 4.2. Dataset

The dataset can be divided into three parts, i.e., training, validation, and testing. The training and validation datasets are used in the experiment for feature extraction analysis, i.e., statistical features and the MFCC and PLP coefficients with 23,356 data points. These datasets contain various road conditions (i.e., paved, unpaved, rough, smooth, potholes, and bumps), different suspension systems (i.e., soft or hard), and different speeds (i.e., 20, 40, 60, or 80 km/h). Each paved and unpaved road consists of 11,678 data points.

On the other hand, the testing dataset is used to evaluate the proposed feature extraction methods considering the conditions mentioned above (this part will be described in Section 5). The testing datasets consists of 8908 data points where 6286 data points for paved and 2622 data points for the unpaved road. The distributions of data for both paved and unpaved road were unbalanced due to the experiment for potholes are exists only on the paved road.

### 4.3. Performance Evaluation

The model performance is evaluated according to the accuracy score defined as

(1) $$Accuracy=\frac{TP}{TP+FP+FN+TN}$$

where *TP* is true positive, *FP* is false positive, *FN* is false negative, and *TN* is true negative. The datasets were divided into two classes: label 0 for paved road and label 1 for unpaved road.

To increase the significance of the results, the training and validation datasets were divided into ten subsets by k-fold cross-validation methods [24]. The results presented in Section 5 are the average values obtained by ten k-fold cross-validation data.

## 5. Experiments and Results

In this section, we present two experiments such as the evaluation of three different feature extraction methods, i.e., the statistical features and MFCC and PLP coefficients using training and validation dataset and the experiments using testing dataset. Both MFCC and PLP have been proposed in the speech recognition field. To adapt them to the road condition classification task, we used them with some modifications. The SVM model is applied as the classification model to compare the performance of each feature extraction method. Finally, we discuss the effectiveness of the modified MFCC and modified PLP, which we propose in this study, for the classification task in road monitoring.

### 5.1. The Experiment Using Training and Validation Dataset

#### 5.1.1. Statistical Feature Extraction

**Figure 3:**
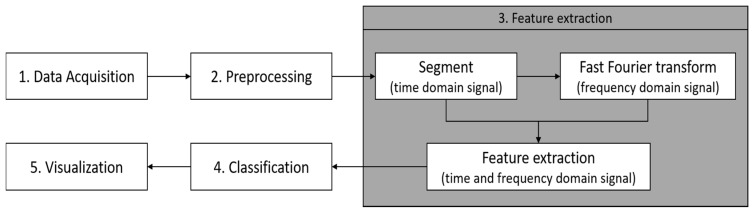
An architecture for statistical feature extraction.

Figure 3 shows the steps used for calculating statistical feature extraction. The filtered signals from the preprocessing step are then sampled into fixed-size sliding windows of 2 s with 20% overlaps. From the time domain signals, the data were also transformed into the frequency domain through a fast Fourier-transform (FFT). For each sample window, a vector of features, such as the mean, variance, standard deviation, mean absolute deviation, maximum, minimum, etc., are obtained for both the time and frequency domain [25].

A total of thirteen statistical methods were used to compute the features in both the time and frequency domains from accelerometer (x, y, z) and gyroscope (x, y, z) signals. Table 3 shows the list of computing statistical feature vectors. The extracted statistical features consist of a total of 130 feature vectors.

**Figure 4:**
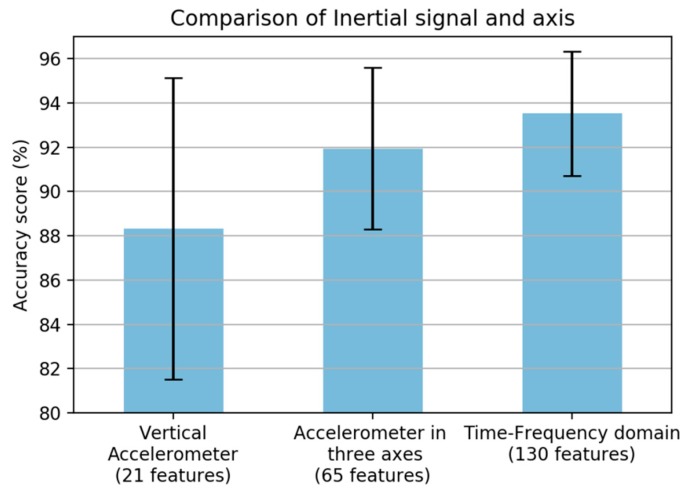
Comparison of inertial sensor and axis.

**Figure 5:**
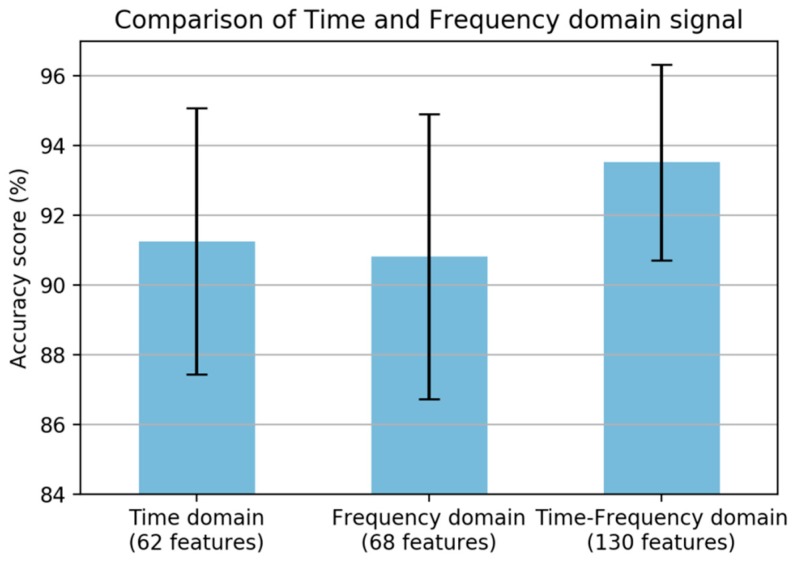
Comparison of statistical feature extraction.

Commonly, some information from only the vertical axis of the accelerometer sensor is used in road condition monitoring. However, vibration will occur in all three axes. Therefore, we adopted in total the three axes of the accelerometer and gyroscope, and the performance was increased significantly once all axes were included, as shown in Figure 4. The main analysis for statistical feature extraction is the comparison of the time and frequency domain features. As shown in Figure 5, the best result was obtained from the time-frequency domain; however, the frequency-domain features obtained a lower result.

#### 5.1.2. Mel Frequency Cepstral Coefficients (MFCCs)

**Figure 6:**
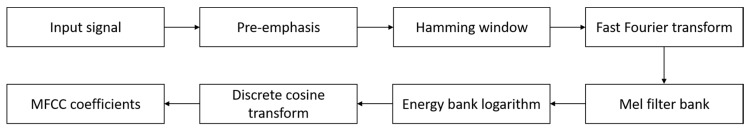
The general Mel Frequency Cepstral Coefficient (MFCC) computation steps.

The Mel Frequency Cepstral Coefficient (MFCC) is a feature widely used in automatic speech recognition. The MFCC technique concentrates resolution analysis at low frequencies [21]. The general workflow of MFCCs is shown in Figure 6.

**Figure 7:**
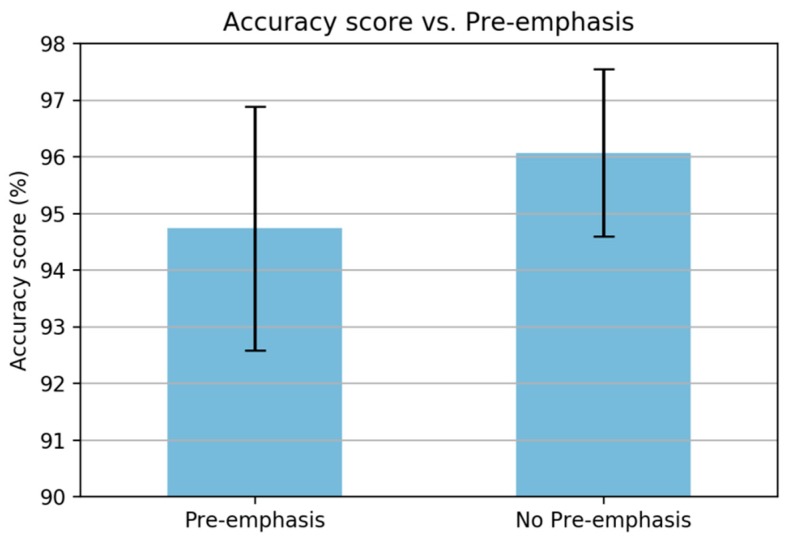
Accuracy score based on pre-emphasis.

There are six steps in computing MFCC coefficients. The first step is amplifying the high frequency on the signal by applying a pre-emphasis filter. San-Segundo et al. [21] described that the pre-emphasis step may be unnecessary because the frequency range in the inertial signal is smaller than the speech signal. This argument is strengthened by our experiments with and without pre-emphasis, as shown in Figure 7. The figure shows that there is an improvement in the accuracy score when removing the pre-emphasis step.

The next step is the application of a Hamming window to split the signal into the short-time scale in order to obtain a good approximation of the frequency spectrum when computing the fast Fourier transform (FFT). Furthermore, the frequency band energy from the FFT signal is computed by applying a mel filter bank (Equation (2)).

(2) $$Mel\left(f\right)=2595*\log\left(1+\frac{f}{700}\right)$$

**Figure 8:**
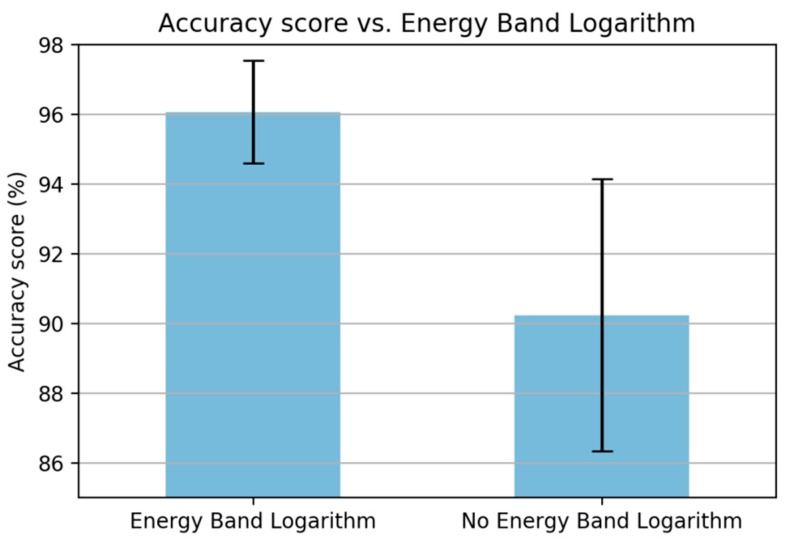
A comparison when removing the energy band logarithm.

The next step is the energy band logarithm which is used to compute the logarithm of the frequency band energy. An experiment was also done to confirm the efficiency of this step, as shown in Figure 8. The figure shows that the performance increases when removing this step.

**Figure 9:**
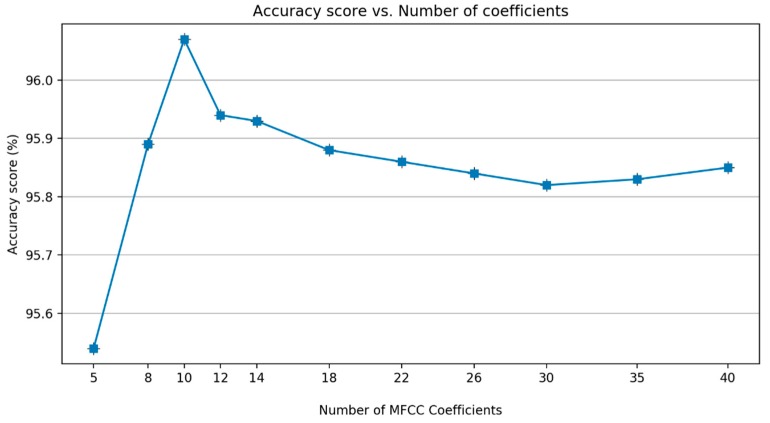
A comparison of the number of MFCC coefficients.

The next step is computing Discrete Cosine Transform (DCT) to compress the frequency information. To determine the number of MFCC coefficients, an additional experiment was carried out. Figure 9 shows that there is a decrease in performance after the coefficient of 10. Therefore, in this study, 10 MFCC coefficients are chosen as the best alternative.

**Figure 10:**
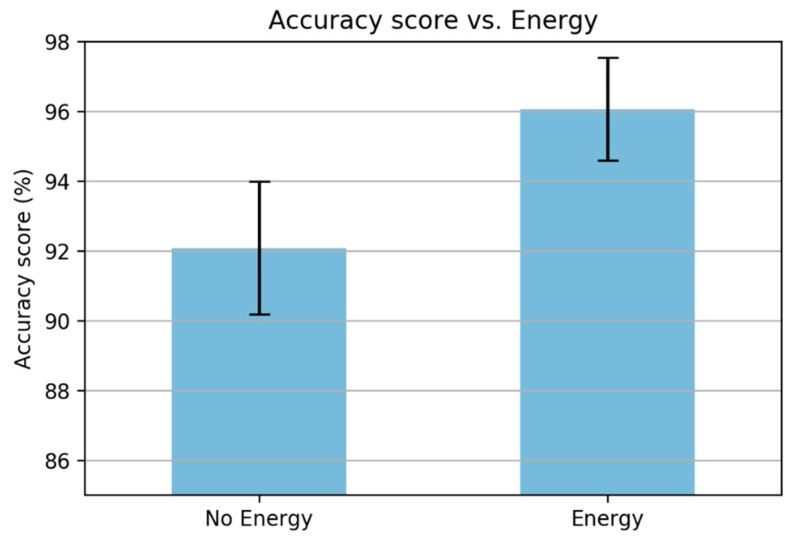
A comparison when removing the energy framed signals.

The next analysis involves adding the energy of the framed signal (Equation (3)). The performance was increased significantly when adding energy of framed signals, as shown in Figure 10.

(3) $$Energy=\overset{n}{\underset{i=1}{\sum }}{\left(x_{i}\right)}^{2}$$

**Figure 11:**
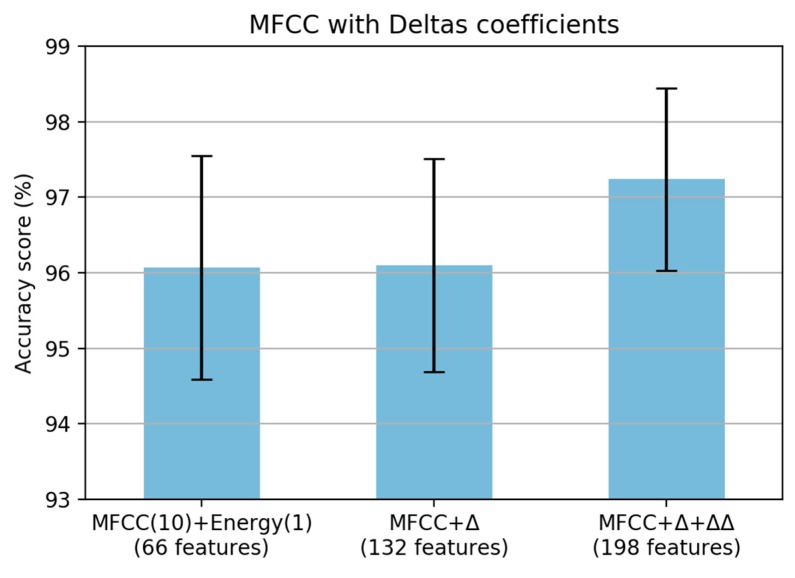
Accuracy score based on MFCC with deltas coefficient.

**Figure 12:**
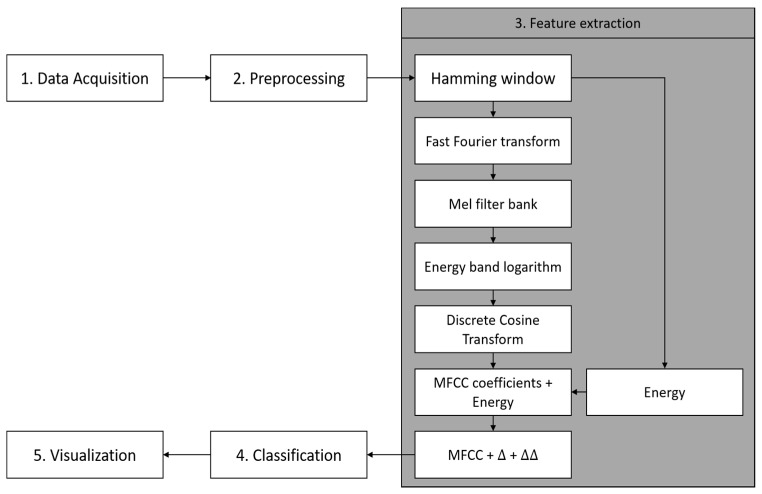
A proposed step for MFCC feature extraction.

The final analysis for MFCC feature extraction is the adding of the deltas (Δ) and delta–deltas (ΔΔ) coefficients. The performance of a speech recognition system can be greatly enhanced by adding time derivatives to the basic static parameters [21]. The deltas coefficient is also known as the differential and acceleration coefficient [26]. The signal derivatives provide information about the signal evolution [21]. The deltas coefficients are defined as follows

(4) $$d_{t}=\frac{\sum_{n=1}^{N}n(C_{t+n}-C_{t-n})}{2\sum_{n=1}^{N}n^{2}}$$

where ($d_{t}$) is a deltas coefficient at time *t* in terms of the static coefficients $C_{t+n}$ to $C_{t-n}$. The typical value of the deltas window size (N) is 2. The same formula is applied to the deltas coefficients to obtain the acceleration coefficients [27]. In this experiment, we added the deltas and delta–deltas coefficient for the proposed MFCC coefficients, i.e., 10 features of MFCC and a feature for the energy. Figure 11 shows that there is almost no change when adding a deltas coefficient with 132 features. However, there was a significant increase in the accuracy score when adding delta–deltas coefficients with 198 features. Finally, the proposed steps of MFCC for paved and unpaved road classification are shown in Figure 12. From the original MFCC, we removed the pre-emphasis step; however, we added the energy of the framed signal and both deltas and delta–deltas coefficients to adapt the paved and unpaved road classification task.

#### 5.1.3. Perceptual Linear Predictive (PLP)

**Figure 13:**
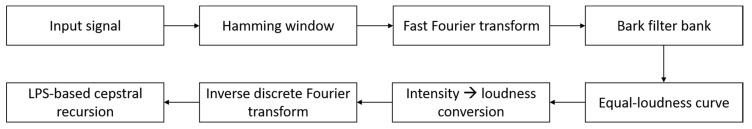
The general Perceptual Linear Predictive (PLP) computation steps.

**Figure 14:**
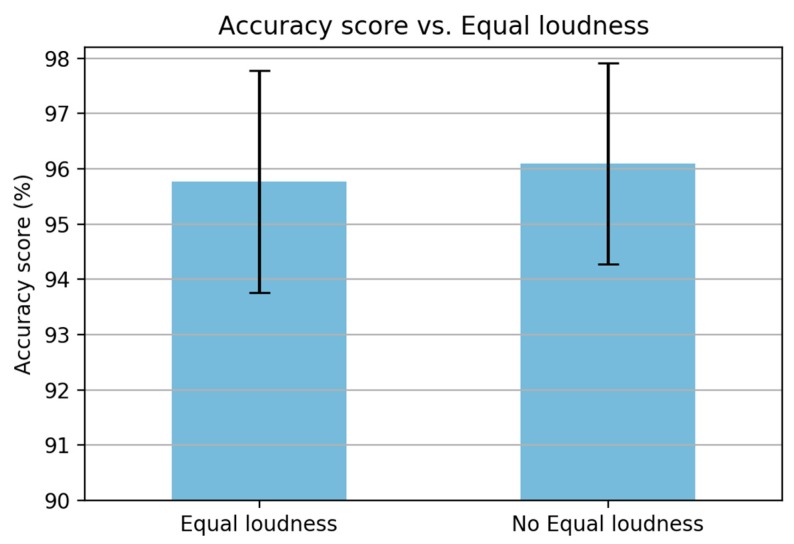
A comparison when removing the equalization in PLP computation.

**Figure 15:**
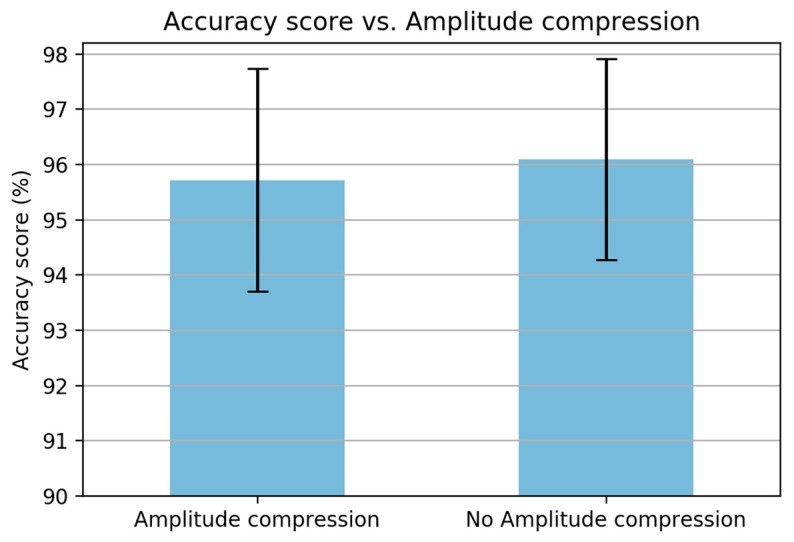
A comparison when removing the compression in PLP computation.

Perceptual Linear Predictive (PLP) is a well-known frequency-based feature extraction method that is widely used in speech recognition. In the PLP technique, several well-known properties of hearing are simulated by practical engineering approximations, and the resulting auditory-like spectrum of speech is approximated by an autoregressive all-pole model [28]. The general workflow of PLP is shown in Figure 13. PLP consists of the following steps. (i) The application of the hamming window to split the signal into a short-time scale to obtain a good approximation of the frequency spectrum when computing the fast Fourier-transform (FFT). (ii) Frequency warp into the Bark scale to find the low frequency resolution (Equation (5)).

(5) $$Bark\left(f\right)=13\arctan\left(0.00076f\right)+3.5\;\arctan\left({\left(\frac{f}{700}\right)}^{2}\right)$$

(iii) The equal loudness curve is used to simulate the sensitivity of hearing about the 40 dB level [28]. This step is similar to the pre-emphasis step in MFCC. Based on the discussion of MFCC in the previous section, the pre-emphasis step may be unnecessary due to the frequency range in the inertial signal being smaller than that in the speech signal. Therefore, to prove this argument, an experiment is carried out by removing the equalization step, as shown in Figure 14. The figure shows that there is a slight improvement when removing the equalization step. Hence, this step is removed to reduce the computational time.

(iv) The next step is the intensity–loudness conversion. This step is an approximation to the power law of hearing by taking the power of 0.33 of the intensity [28,29]. This step is equivalent to the energy band logarithm in MFCC [21]. In the MFCC analysis, the result was decreased when removing the energy band logarithm. However, in contrast, in PLP computation, the result is increased when excluding the amplitude compression (Figure 15). (v) The last step prior to the PLP coefficients is application of an inverse discrete Fourier-transform (IDFT). (vi) The PLP coefficients are obtained by applying a linear predictive coding (LPC).

**Figure 16:**
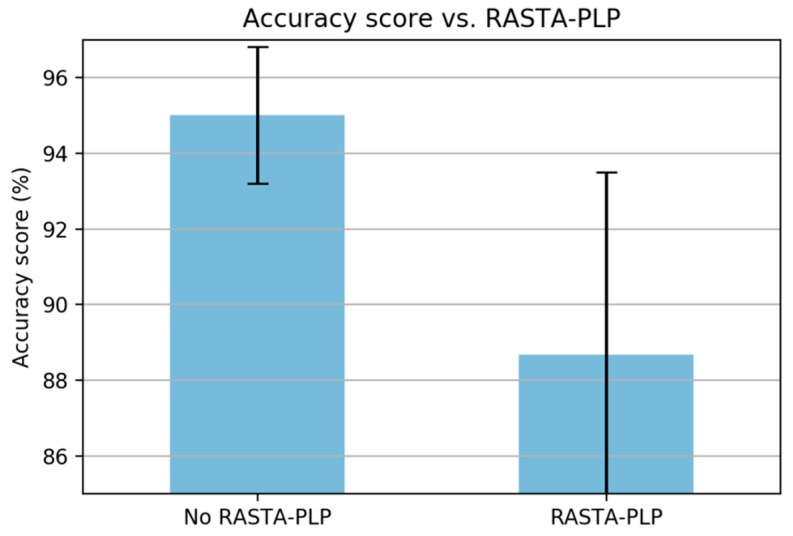
A comparison of the added RASTA filter.

In PLP computation, it is known that a relative spectra (RASTA) filter can improve the recognizer performance [21,30]. The RASTA filter is applied to suppress slow and quick changes of the signal [30]. In this study, the RASTA filter was unable to improve the classification performance (Figure 16) due to the slow and fast changes in the signal being part of the anomalous pattern that needs to be taken into account, i.e., breaking event, potholes, and bumps (for further investigation of anomaly detection on the classified paved roads).

**Figure 17:**
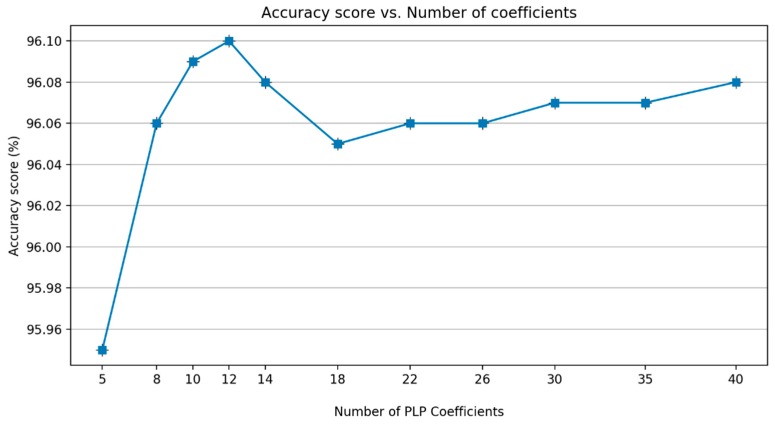
A comparison of the number of PLP coefficients.

Similar to MFCC, the next analysis involved defining the number of PLP coefficients. Figure 17 shows that there is a decrease in performance after the coefficient of 12. Therefore, a coefficient of 12 is defined as the best alternative. The energy of the framed signal was added based on the experience in the MFCC experiment.

**Figure 18:**
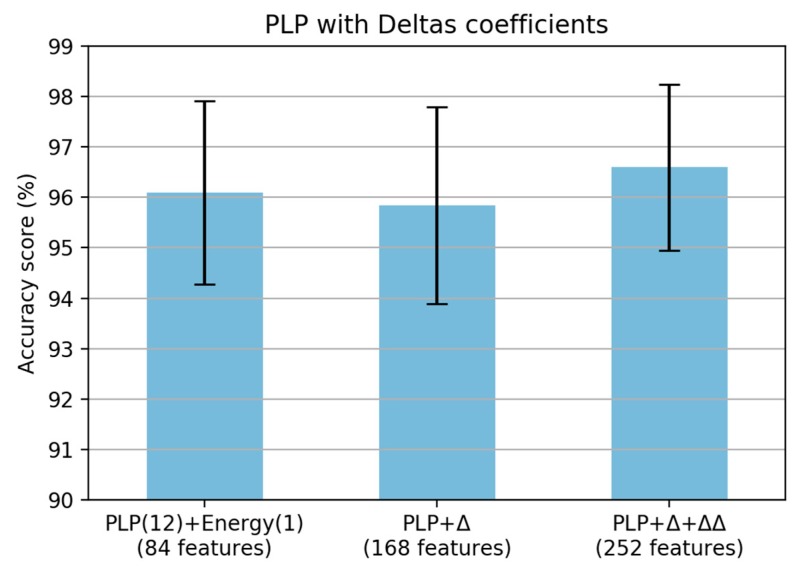
Accuracy score based on PLP with deltas coefficient.

**Figure 19:**
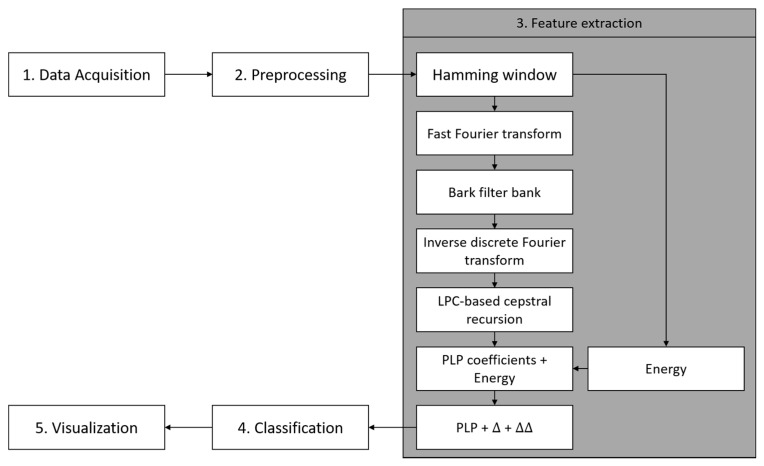
A proposed step for PLP feature extraction.

The final analysis for PLP feature extraction is the addition of deltas and delta–deltas coefficients. When adding a single deltas coefficient, the performance was decreased; however, it was improved when adding a double deltas coefficient in the PLP computation (Figure 18). Finally, the proposed PLP computation for the smartphone inertial signal is shown in Figure 19. From the original PLP, we added the energy of the framed signal and delta–deltas coefficients; however, we removed the equal-loudness curve and amplitude compression to adapt the paved and unpaved road classification task.

### 5.2. The Experiment Using Test Dataset

To ensure that the use of advanced frequency-based feature extraction methods are useful in a road condition classification problem, i.e., paved and unpaved road, we tried to evaluate the proposed feature extraction methods using a test dataset, which considers various road conditions (i.e., paved, unpaved, smooth, rough, potholes, and bumps), different vehicle suspension system (i.e., soft or hard), and different vehicle speeds (i.e., 20, 40, 60, or 80 km/h). In addition, the test dataset was different from the training dataset. In this study, we have not compared the result based on the Smartphone sensor yet. On the other hand, we found that there are up to ~5% of errors on sensor values. We have not evaluated the influences of such errors on classification results yet. We are trying to find an automatic way to calibrate the sensor values using initial part of recording data.

The test dataset is divided into three parts, i.e., suspension, road condition, and speed. The first part is the suspension, which consists of hard and soft vehicle suspension systems. The second part is the road condition, which consists of several road conditions, i.e., potholes and bumps, the roughness of a paved road, and the smoothness of an unpaved road. The last part is the speed, which consists of different vehicle speeds on the same road segment, i.e., 20, 40, 60, or 80 km/h.

The overall result for the test dataset is shown in Table 4. The table shows that the use of advanced frequency-based features, i.e., MFCC and PLP, achieved a better result than the statistics-based features.

The following describes the results for the suspension, road condition, and speed.

#### 5.2.1. Soft vs. Hard Suspension

To confirm the influence of vehicle suspension type on the classification result, we conducted experiments using several different vehicle suspension systems on the same road segment, i.e., soft and hard suspension. The difference between soft and hard suspension is the sensitivity in producing an amplitude response where the soft suspension produces a higher amplitude than the hard suspension. Table 5 shows that the use of soft suspension affects the performance of the classification due to the soft suspension producing a high amplitude even though the vehicle passes a roughly paved road. Therefore, the signal showed almost the same characteristics as the smooth unpaved road. Therefore, the soft suspension achieved a lower result than the hard suspension in all feature extraction methods. Nevertheless, the frequency-based features could significantly overcome the use of different suspension systems. As shown in Table 5, the frequency-based features, i.e., MFCC and PLP, obtained a better result than the statistical features, especially for the use of a soft suspension.

#### 5.2.2. Pothole and Rough Paved vs. Smooth Unpaved

To differentiate a pothole, rough paved road, and smooth unpaved road, an experiment was conducted. The test dataset used in this experiment consisted of potholes, bumps, rough roads (paved) and smooth roads (unpaved). This experiment was conducted to ascertain how accurate the system was in distinguishing the condition of the roads mentioned earlier (for further investigation of pothole and bump detection, as well as roughness estimates). Table 6 shows that the statistical method achieves poor results compared to MFCC and PLP, especially for the pothole and rough road (paved). On the other hand, for the unpaved road, the three methods produce very good results.

#### 5.2.3. Different Vehicle Speed

In a road segment of both paved and unpaved roads, four different vehicle speeds were conducted: 20, 40, 60, or 80 km/h. Table 7 shows that the statistical method is less accurate for paved when compared to the MFCC and PLP methods. In contrast, on the unpaved road type, all methods achieve a good result.

### 5.3. Results of Visualization

**Figure 20:**
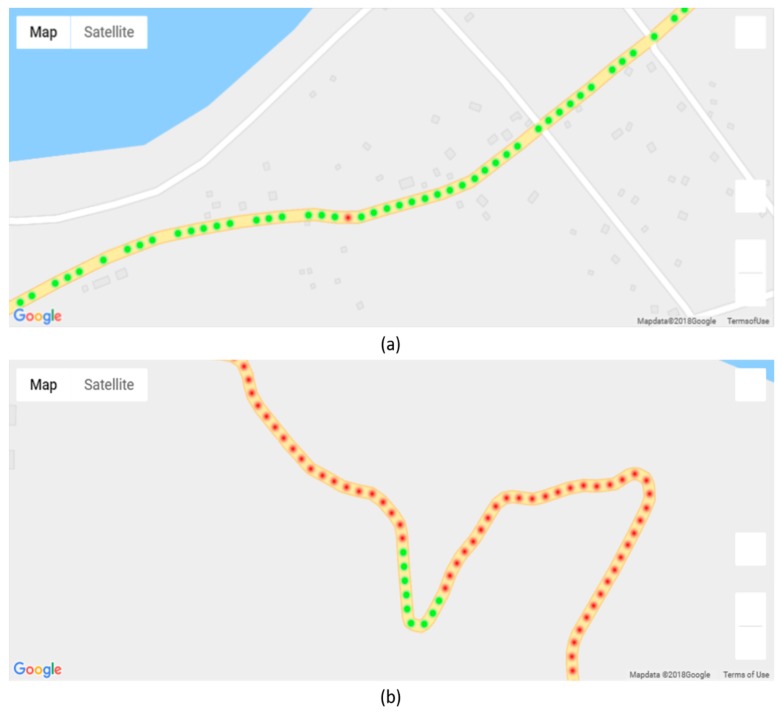
Result of maps visualization: (**a**) paved roads and (**b**) unpaved roads.

As discussed earlier, the results of classification can be visualized based on a map. Figure 20 shows a geographical visualization of our result on a map provided by Google Maps. Each point represents an aggregation of classification results, where the green points represent paved road and red points represent the unpaved road. As shown in Figure 20a,b, there is misclassification in both the paved and unpaved road sections.

## 6. Discussion

Based on this series of analysis using training and validation datasets, we concluded that both MFCC and PLP coefficients are very effective in the feature extraction step for the task of classifying paved and unpaved roads with improved accuracy, compared to statistical feature extraction. Basically, both MFCC and PLP were designed for speech recognition; however, these methods have also been used in other research areas, such as human activity recognition (HAR) [19,20,21] and environmental sound recognition [31]. Inspired by the workflow analysis for both MFCC and PLP described in the article [21], we propose a final computation procedure for paved and unpaved road classification, as shown in Figure 12 (MFCC) and Figure 19 (PLP). In the analysis, the pre-emphasis signal was eliminated due to the frequency range in the inertial signal being smaller than that in the speech signal. As a result, there were improvements in performance observed when removing the pre-emphasis step in both MFCC and PLP computation (Figure 7 and Figure 14). Taking the logarithm of mel filter bank in MFCC computation produces large variations in energy; therefore, removing the energy band logarithm step provokes a decrease in performance (Figure 8), hence this step is maintained. Contrarily, removing a cube root of the bark filter bank in PLP computation provides a small improvement due to the energy variations being not much different (Figure 15); hence, this step is removed. One of the advantages of MFCC and PLP is the delta coefficients. Our analysis proved that adding a delta–deltas coefficient can improve performance (Figure 11 and Figure 18). In addition to the deltas coefficient, a RASTA filter is also believed to be able to improve performance significantly, as evidenced in the article [21]. The RASTA is a filter that will suppress slow and quick changes of a signal. However, in this study, it was shown that applying the RASTA filter showed a decrease in performance (Figure 16) due to fast and slow changes in the signals being also taken into account, i.e., high speed when passing through potholes or bumps. In addition, the MFCC and PLP are effectively implemented in road condition classification due to both techniques efficiently compress the frequency information. In the next section, only the proposed computation for Statistical, MFCC, and PLP will be used in the experiments over test dataset.

On the other hand, the experiments over test datasets, it can be concluded that there is a significant influence on the use of suspension types (i.e., hard and soft). The result shows that in the classification for the unpaved road, all three methods achieved a better result rather than classification for the paved road. This result is due to the vehicle with soft suspension producing high amplitude when passing through a rough section of paved road. This signal has similar characteristics to the unpaved road signal. Nevertheless, the MFCC and PLP methods achieved better results compared to the statistical method in all experiments. 

It is important to note that the frequency-based features, i.e., MFCC and PLP, worked well in this task due to the following aspects; the first aspect is the energy concentration in the lower frequency range. The frequency ranges for speech and inertial signals are different; however, the energy of both signals is concentrated in the lower frequency range [21]. The analysis was done by applying the warping schema, i.e., mel and Bark scale. The second aspect is a cepstral analysis, which is used to extract the important pattern [21]. The last aspect is taking the energy of the frame.

## 7. Conclusions

This work was conducted to improve the accuracy of paved and unpaved road classification by making a detailed investigation of the feature extraction method. Signals were extracted from the acceleration and gyroscope sensors in a common smartphone. In this study, data collection was conducted on the East Timor road network, which consists of different road conditions, i.e., smooth, rough, potholes, and bumps, on a paved road and an unpaved road. The data collection involved four different vehicles, i.e., Toyota Land Cruiser Prado, Mitsubishi Pajero, Toyota Hi-Lux, and Daihatsu Xenia, as well as different smartphone devices, i.e., Samsung Galaxy S8, Samsung Galaxy A5, and Asus ZenFone.

To improve the performance of classification, this study proposes a well-known frequency-based feature extraction, i.e., MFCC and PLP coefficients, which are commonly used in speech signal processing. For a final proposal of both MFCC and PLP computations, several experiments are conducted. In the analysis, some steps are discarded, i.e., pre-emphasis of MFCC and equal-loudness curve and amplitude compression of PLP. On the other hand, the energy of the framed signal was added to the computation. The performance was significantly improved when adding delta–deltas coefficients to the computation step for both MFCC and PLP. 

Other experiments are also conducted for the road conditions. In the experiments, the test dataset was divided into three parts, i.e., road, suspension, and speed. The experimental results concluded that there was a significant influence on the use of suspension type and vehicle speed for the road surface conditions monitoring. In particular, the statistics-based method achieved poor results compared to the MFCC and PLP method. Finally, based on all experiments either using validation or test datasets, one can conclude that the use of frequency-based features overcomes the performance of statistics-based features. 

Our future work will focus on pothole and bump detection, pothole size estimation, and roughness estimation for both paved and unpaved roads based on roughness indices.

## Figures and Tables

**Table 1:** Previous research on smartphone base in road surface monitoring.

References	Applications	Sensors	Features	Methods
Tai et al. [10]	Anomaly detection	Accelerometer & GPS	Mean, standard deviation, accelerometer range, maximum, minimum, and mean speed	SVM
Bhoraskar et al. [12]	Traffic and road condition estimation	Accelerometer & GPS	Mean, standard deviation, and difference metric	K-means and SVM
Tomiyama et al. [13]	Road roughness estimation	Accelerometer & GPS	Velocity and slope profile	International Roughness Index (IRI)
Seraj et al. [11]	Anomaly detection	Accelerometer, gyroscope & GPS	1. Time domain (mean, standard deviation, variance, peak to peak, root mean square, zero crossing rate, mean of absolute value, correlation of all axis, tilt angles, wave form length and signal magnitude area)	SVM
			2. Frequency domain (mean, median, and energy).	
			3. Wavelet transform	
Masino et al.	Road damages detection	Accelerometer, gyroscope & GPS	Wavelets transform, distance between trajectories, and PCA	SVM
Kumar et al. [16]	Road roughness monitoring	Accelerometer & GPS	Mean of absolute value	Fuzzy classifier
Singh et al. [17]	Road surface monitoring	Accelerometer & GPS	Speed and distance matrix	DTW
Cabral et al. [7]	Paved and unpaved road classification and anomaly detection	Accelerometer, gyroscope & GPS	Mean, standard deviation, variance, mean absolute deviation, maximum, minimum, root mean square, signal magnitude area, interquartile range, correlation coefficients of axis, energy, entropy, and skewness	SVM, HMM, ResNet, k-NN & DTW

**Table 2:** Parameters setting for the support vector machine (SVM) model.

Features Extraction Method	Parameters				
	Kernel	C		Gamma	
	Exp. 1 & 2	Exp. 1	Exp. 2	Exp. 1	Exp. 2
Statistical	Radial Basis Function	100	5	0.001	0.0001
MFCC + Δ + ΔΔ		50		0.00001	
PLP + Δ + ΔΔ		100		0.0001	

**Table 3:** List of computing feature vector for statistical method.

Features	Time Domain	Frequency Domain	Time-Frequency Domain
Mean	6	6	12
Variance	6	6	12
Standard Deviation	6	6	12
Mean absolute deviation	6	6	12
Maximum	6	6	12
Minimum	6	6	12
Root mean square	6	6	12
Signal magnitude area	2	2	4
Interquartile range	6	6	12
Correlation coefficient	6	0	6
Energy	0	6	6
Entropy	6	6	12
Skewness	0	6	6

**Table 4:** An overall result of test dataset.

Features Extraction	Number of Features	Model Accuracy (%)
Statistical	130	86.23
MFCC + Δ + ΔΔ	198	98.07
PLP + Δ + ΔΔ	252	96.80

**Table 5:** Results from different suspension systems.

Features Extraction	Number of Features	Model Accuracy (%)	
		Soft	Hard
Statistical	130	81.67	97.53
MFCC + Δ + ΔΔ	198	97.96	99.57
PLP + Δ + ΔΔ	252	96.92	99.32

**Table 6:** Results from different road conditions.

Features Extraction	Number of Features	Model Accuracy (%)		
		Pothole ^1^	Rough ^1^	Smooth ^2^
Statistical	130	55.28	88.44	88.88
MFCC + Δ + ΔΔ	198	96.57	98.82	96.38
PLP + Δ + ΔΔ	252	85.68	97.66	97.55

^1^ Paved, ^2^ Unpaved.

**Table 7:** Results from different vehicle speeds.

Features Extraction	Number of Features	Model Accuracy (%)	
		Paved	Unpaved
Statistical	130	85.43	99.69
MFCC + Δ + ΔΔ	198	98.88	98.71
PLP + Δ + ΔΔ	252	99.61	99.06
